# Estrogen deficiency due to type 2 diabetes induced hyposalivation in female mice by promoting inflammation in the salivary glands

**DOI:** 10.1007/s10266-025-01158-6

**Published:** 2025-07-29

**Authors:** S. E. Cifuentes-Mendiola, A. L. García-Hernández, N. Cruz-Mendoza, I. O. Pérez-Martínez, I. X. Cruz-García

**Affiliations:** 1 Osteoimmunology Section, Laboratory of Dental Research, Clínica Universitaria de Salud Integral Almaraz, FES Iztacala UNAM, Av. Jiménez Gallardo SN, San Sebastián Xhala, CP 54714 Cuautitlán Izcalli, Mexico State Mexico; 2 Section of the Neurobiology of Oral Sensations, Laboratory of Dental Research, FES Iztacala UNAM, Cuautitlán Izcalli, Mexico State México

**Keywords:** Estrogen, Salivary Glands, Type two diabetes, Hyposalivation, Female mice, Estradiol

## Abstract

Estrogen deficiency is related to salivary gland dysfunction, and the prevalence of hyposalivation in women with type 2 diabetes (T2D) is higher than the general population; nevertheless, the contribution of estrogen to hyposalivation in T2D is unclear. Four-week-old female C57BL/6 mice were divided into four groups (*n* = 6): control, ovariectomized (OVx), T2D, and OVx–T2D. T2D was induced by a combination of a high-carbohydrate diet and low-dose streptozotocin. Estrogen deficiency was induced at 8 weeks of age by OVx. At 20 weeks of age, stimulated saliva secretion and quality, blood glucose, and serum 17β-estradiol levels were determined, and the parotid and submandibular glands were obtained, histologically processed, and analyzed for histomorphometry; fibrosis development; and the expression of interleukin (IL)-1, IL-6, IL-10, IL-17, tumor necrosis factor (TNF)-α, and α-amylase. Compared with the control group we found a decrease in the 17β-estradiol concentration, an increase in body weight, a decrease in saliva secretion with alterations in its quality, a decrease in parotid and submandibular glands number of acini and α-amylase, acinar hypertrophy and fibrosis and increase in expression of IL-1, IL-6, IL-17, and TNF-α with a decrease in IL-10 in OVx, T2D, and OVx–T2D groups. In conclusion, T2D reduces 17β-estradiol levels comparable to OVx, and loss of estradiol results in hyposalivation and histological alterations through the establishment of a proinflammatory microenvironment in female mice parotid and submandibular glands.

**Supplementary Information:**

The online version contains supplementary material available at 10.1007/s10266-025-01158-6.

## Introduction

Type 2 diabetes mellitus (T2D) is a worldwide epidemic with 482.94 million active cases, and with a mortality rate of 6.7 million deaths in 2021 [1]. The course of T2D is characterized by the occurrence of insulin resistance and, accordingly, a relative deficiency in the secretion of this hormone, which is accompanied by hyperglycemia [2]. T2D is associated with reduced circulating levels of estradiol with elevated levels of follicle-stimulating hormone [3], leading to premature postmenopause, which promotes abdominal adipose tissue accumulation and reduces insulin sensitivity [4]. These hormonal changes can play a role in T2D pathophysiology and severity of related complications [5].

Among the main oral complications of T2D is hyposalivation [6], which is a decrease in saliva flow [7] and leads to changes in the oral and intestinal microbiota; increased incidence of caries and oral infections; digestive problems; polydipsia; dysgeusia; fissures and wounds on the lips; burning mouth syndrome; and angular cheilitis [7, 8]. The prevalence of hyposalivation in T2D patients is high, ranging from 41 to 65% [9–12], and it has been estimated that in North America, hyposalivation is present in at least 10 million indivpiduals with T2D [13]. Additionally, poor saliva quality has been reported in individuals with T2D [14, 15]. Notably, a higher prevalence of hyposalivation has been reported in women with T2D than in men with T2D [10, 16].

Hyposalivation in T2D may be due to damage to the parenchyma of the salivary glands, alterations in glandular microcirculation, dehydration, and alterations in glycemic control [12]. However, one potential mechanism for the development of hyposalivation with T2D is estrogen deficiency since estrogen receptors are expressed in the salivary glands [17], and it has been reported that estrogen deficiency is associated with salivary gland dysfunction and is considered a key factor in disorders of these glands [13]. A higher prevalence of hyposalivation has been reported in postmenopausal women than in premenopausal ones [18], and hormone replacement therapy has been shown to increase salivary flow and decrease the sensation of dry mouth in postmenopausal women [17].

Reduction of estrogen has been associated to inflammation increase, which favor chronic low-grade inflammation through an increase in inflammatory cytokines, such as IL-1β, IL-6 and TNF-α and a decrease in immunoregulatory mechanisms [19, 20], which triggers the development of hyposalivation and xerostomia, as observed in patients with metabolic syndrome [21] and in postmenopausal patients [22]. However, to our knowledge, this has not been studied in the context of T2D.

Therefore, in this work, we determined whether T2D leads to changes in estrogen levels and whet9her these alterations contribute to the induction of hyposalivation, histomorphometric alterations and the establishment of an inflammatory microenvironment in the parotid and submandibular salivary glands in female mice with T2D.

## Materials and methods

### Animals

We used 4-week-old female C57BL/6 mice (CINVESTAV, IPN, México), which were divided into four experimental groups: control, estrogen deficiency due to ovariectomy (OVx), T2D, and T2D with OVx (OVx–T2D). All the groups included six mice per group. The mice were housed under bioterium conditions in polysulfone cages with a gentle-air filter vent cage top (RC71M-UD, VTA71D-UD-FT Alternative Design Manufacturing and Supply, Inc.) at 22 °C, 45% humidity, and a 12-h photoperiod with free access to food and liquid. At 20 weeks of age, the mice were euthanized via an anesthesia overdose (sodium pentobarbital, Aranda), and both the parotid and submandibular salivary glands and blood serum were obtained and stored at − 80 °C until use. The salivary glands were weighed, and the average weight of each pair was determined. All procedures were performed in accordance with the recommendations of the Mexican Official Standard NOM-062-ZOO-1999, the “American Veterinary Medicine Association” (2022), and the approval of the University Ethics Committee (FES-Iztacala, UNAM, CE/FESI/112022/1566).

### T2D induction

Previously, a T2D mouse model was developed and standardized in the laboratory [23]. The model consists of the administration of a high-calorie diet rich in carbohydrates throughout the experiment to induce insulin resistance due to obesity and by inducing a relative deficit in insulin secretion with low doses of streptozotocin (STZ, Sigma‒Aldrich, #18883-66-4) at 10 weeks of age (50 mg/kg of body weight on the first day and 25 mg/kg of body weight on days 2–7). The fasting blood glucose level was determined in blood from the caudal vein with a commercial glucometer (OneTouch Select Plus). The mice were considered diabetic when their blood glucose concentrations were greater than 250 mg/dL [23–25]. The mice in the control group were fed a standard diet (Tekland 2018, ENVIGO).

### Estrous cycle homogenization

To avoid variations in estrogen levels, at six weeks of age, the estrous cycle was homogenized through artificial induction of estrus with the subcutaneous administration of estradiol benzoate (10 μg/100 g of body weight). Oeffler, México) and progesterone (0.5 mg/100 g of body weight. Zoetes, México), as previously reported [26].

### Ovariectomy

At eight weeks of age, both ovaries were removed, or sham surgery was performed under 1.5% isoflurane inhalation anesthesia. A bilateral incision was made between the last rib and the pelvis and then an incision was made in the muscle with blunt scissors. Both ovaries were removed with hemostatic forceps and an absorbable suture thread (chromic gut 3.0) and closed the incisions with 6-0 absorbable silk sutures. The procedure was carried out based on a previous report [27]. After the surgical procedure, all the mice were treated with antibiotics (enrofloxacin, 10 mg/kg; Senosiain) and analgesics (meloxicam, 5 mg/kg; Holland) for three consecutive days.

### Quantification of serum 17β-estradiol

17β-estradiol concentrations were determined in blood serum obtained at the end of the experiment by ELISA assay with the commercial “Estradiol ELISA Kit” (Cayman Ch. #582701) according to the manufacturer's instructions.

### Salivary flow assay

Mice were anesthetized by inhalation of 1.5% isoflurane and saliva secretion was stimulated by administration of 40 µL of 2% pilocarpine intraorally for three minutes [27]. After this time, sterile cotton balls were introduced into the oral cavity for saliva absorption for 10 min, and to recover saliva, the cotton balls were centrifuged in Eppendorf tubes at 8000 × *g* for 3 min according to a previous report [28]. Once the saliva was obtained, the total volume was measured with a micropipette.

To determine saliva quality, the weight of the saliva was obtained to determine the density (m/v), pH was determined with test strips, and protein content by the Bradford method. In addition, α-amylase expression was determined by western blotting. 7 µL of saliva was used to separate proteins by sodium dodecyl sulfate–polyacrylamide gel electrophoresis (SDS‒PAGE) on a 10% polyacrylamide gel. Then, the proteins were transferred to a nitrocellulose membrane which was stained with Ponceau red to verify protein integrity and was used as loading control (Fig. S1). Nonspecific binding was blocked with 6% nonfat dry milk. Then, the membranes were incubated overnight with a mouse anti-α-amylase antibody (1:1000, Santa Cruz Biotechnology, #sc-46657) at 4 °C. Then, the membranes were incubated with a rabbit antimouse IgG antibody conjugated to horseradish peroxidase (HRP, Bioss antibodies, #bs-0296R-HRP) for 2 h at room temperature. The binding was detected by chemiluminescence with the SuperSignal West Pico PLUS Chemiluminescent Substrate Kit (Thermo Scientific, #34580). Images of the blots were obtained to quantify the expression of α-amylase (55 kDa) with ImageJ software.

### Histological analysis

Parotid and submandibular salivary glands were fixed in paraformaldehyde, dehydrated in increasing alcohol, cleared in xylene, and embedded in paraffin (Paraplast plus, Sigma-Aldrich #125387-89-5). Longitudinal serial sections of 5 μm thickness were obtained from parotid and submandibular salivary glands with a microtome). Sections were routinely stained with hematoxylin and eosin (H and E) or with a double stain with fast green/Sirius red to visualize collagen fibers [29] or by immunohistochemistry (IHC) to detect the expression of α-amylase and the cytokines interleukin (IL)-1β, IL-6, IL-10, IL-17, and tumor necrosis factor (TNF)-α with the following antibodies: anti-α-amylase (Santa Cruz Biotechnology, #sc-46657), anti-IL-1β (Santa Cruz Biotechnology, #sc-32294), anti-IL-6 (Santa Cruz Biotechnology, #sc-32296), anti-IL-10 (Santa Cruz Biotechnology, #sc-365858), anti-IL-17 (Santa Cruz Biotechnology, #sc-374218), and anti-TNF-α (Santa Cruz Biotechnology, #sc-130348). The SS Polymer-HRP/DAB Detection Kit (Biogenex, #QD400-60KE) was used to detect protein signals and for negative control (Fig. S2), according to the manufacturer’s specifications.

For analysis, four fields from two different sections per salivary gland were selected for all stained sections, and micrographs were taken at 400 × magnification. All micrographs were taken from the same mouse and region per group. For histomorphometric analysis, each micrograph was divided into four 100 µm^2^ quadrants, and the number and thickness of salivary acini, the number of acinar cells, and the number and thickness of striated or convoluted granular ducts were quantified. The results for each quadrant were averaged, and the results from all the micrographs obtained from each gland were subsequently averaged. For Fast Green/Sirius Red-stained sections and IHC, the percentage of expression in each image was determined; then, the results for each gland and mouse were averaged. All analyses were performed with ImageJ software.

### Statistical analysis

All experimental groups had an “n” of 6 mice per group. Data distribution was analyzed with the D’agostino-Pearson and Anderson–Darling tests. Significant differences between experimental groups were determined by analysis of variance (ANOVA), followed by a Tukey post hoc test. For percentage data analysis, Kruskal‒Wallis nonparametric analysis was performed followed by Dunn’s post hoc test. In all cases, we used GraphPad Prism 9 software and considered statistically significant differences if *p* ≤ 0.05.

## Results

### T2D decreases estradiol, which in turn deteriorates metabolic health

**Fig. 1:**
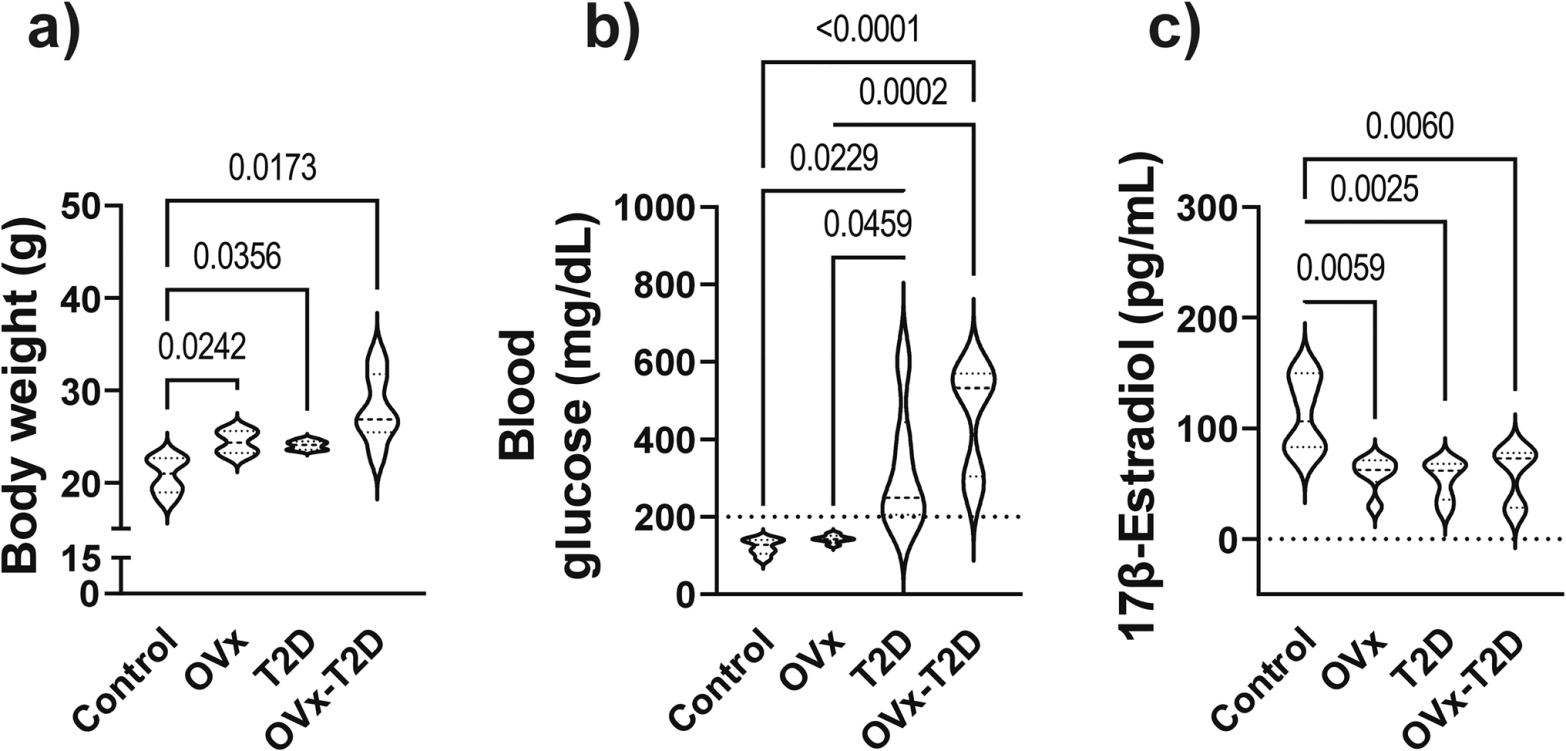
Type 2 diabetes causes decreased estradiol, and decreased estradiol negatively affects metabolic health in female mice with or without type 2 diabetes. **a** Results of body weight determination, **b** blood glucose concentration, and **c** 17β-estradiol concentration in the four groups at 20 weeks of age. The graphs present the means ± SEMs. The brackets express statistical significance, and the *p* value is shown

Compared with those in the control group, the mice in the OVx group had a significantly increased body weight and significantly decreased estradiol concentration, with no changes in the blood glucose concentration (Fig. 1a, b, c). Compared with the control group, the T2D group presented significant increases in body weight and blood glucose concentrations (Fig. 1a and b), with markedly decreased estradiol concentrations that were similar to those of the OVx group (Fig. 1a, b, c). The OVx–T2D group displayed significantly higher body weight and blood glucose, and significantly lower estradiol levels compared with the control group, similar to the OVx and T2D groups (Fig. 1a, b, c).

### Estrogen deficiency in healthy and T2D female mice reduces saliva secretion and quality

**Fig. 2:**
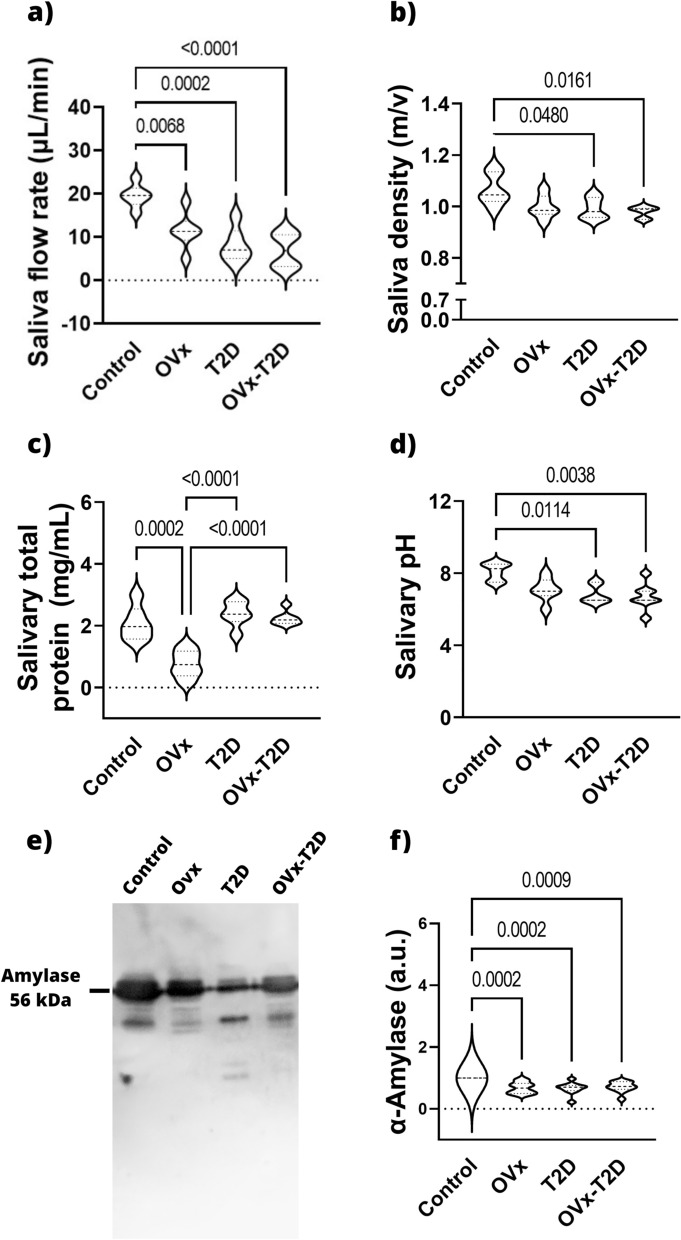
Estrogen deficiency in healthy and T2D female mice reduces saliva secretion and quality*.* The results obtained from the four experimental groups are shown for **a** the pilocarpine-stimulated salivary flow rate (µL/min), **b** the stimulated saliva density, **c** the salivary protein concentration obtained by the Bradford method, **d** the salivary pH and **e**, **f** α-amylase expression in the saliva obtained by western blot. The graphs present the means ± SEMs. The brackets express statistical significance, and the *p* value is shown

Salivary flow analysis revealed that, compared with those in the control group, the total amount of stimulated saliva secreted by the mice in the OVx, T2D, and Ovx–T2D groups was significantly lower, and this decrease was similar in these three experimental groups (Fig. 2a).

We analyzed salivary pH, density, protein content, and α-amylase expression as quality parameters [30] (Fig. 2b, c, d, e, f). As compared to control group, the OVx group presented a significant decrease in total protein concentration and salivary α-amylase (Fig. 2c, e and f). The T2D group presented a significant decrease in salivary pH and α-amylase expression (Fig. 2d, e, f), and similar amounts of protein in saliva to those in the control group (Fig. 2c). The OVx–T2D group presented a significant decrease in pH, salivary density and α-amylase expression in saliva, with a protein concentration similar to that of the control group (Fig. 2b, c, d, e, f); in all cases, we found no significant difference between the T2D and OVx–T2D groups.

### Estrogen deficiency in healthy and T2D female mice causes decreased acini number, acinar hypertrophy, fibrosis, and decreased α-amylase in the parotid and submandibular glands

First, we analyzed the weights of the parotid and submandibular glands. We found no differences in the weights of the parotid glands (Table 1), but the weights of the submandibular glands were greater in the OVx, T2D, and OVx–T2D groups as to control group (Table 2).

**Table 1:** Results of histomorphometric analysis and cytokine expression in the parotid glands

Parotid gland				
Parameter/group	Control	OVx	T2D	OVx–T2D
Histomorphometry				
Parotid gland weight (g)	0.0065 ± 0.003	0.0116 ± 0.001	0.0142 ± 0.002	0.0167 ± 0.003
# Acini (100 μm^2^)	14.69 ± 0.974	8.91 ± 0.945^***aa***^	6.74 ± 0.741^***aaaa***^	8.64 ± 1.016^***aaa***^
Acini thickness (μm)	15.09 ± 0.784	23.00 ± 2.025^***aa***^	26.85 ± 0.834^***aaa***^	24.57 ± 1.920^***aa***^
# Acinar cells	5.97 ± 0.161	5.95 ± 0.391	5.943 ± 0.232	5.738 ± 0.273
# Striated ducts (100 μm^2^)	0.62 ± 0.168	0.55 ± 0.171	0.70 ± 0.057	0.71 ± 0.125
Striated ducts thickness (µm)	20.28 ± 1.759	20.37 ± 1.029	22.72 ± 2.086	20.92 ± 1.131
Cytokines				
IL-1 (%)	3.70 ± 1.038	24.69 ± 6.203^***a***^	26.34 ± 3.723^***a***^	38.79 ± 6.539^***aaa***^
IL-6 (%)	11.18 ± 3.821	33.59 ± 4.364^***a***^	40.45 ± 5.569^***aaa***^	31.30 ± 4.174^***a***^
IL-10 (%)	37.65 ± 6.211	19.96 ± 1.946^***a***^	12.82 ± 2.92^***aa***^	10.70 ± 5.048^***aa***^
IL-17 (%)	6.70 ± 3.093	40.90 ± 10.810^***aa***^	35.49 ± 1.368^***a***^	30.65 ± 5.884
TNF-α (%)	30.73 ± 3.996	58.81 ± 6.824^***a***^	55.56 ± 5.395^***a***^	58.81 ± 6.180^***a***^

*a* vs. control, *b* vs. OVx,u *c* vs. T2D, *d* vs. OVx–T2D. *a* < 0.05, *aa* < 0.01, *aaa* < 0.005, *aaaa* < 0.001

**Table 2:** Results of histomorphometric analysis and cytokine expression in the submandibular glands

Submandibular gland				
Parameter/Group	Control	OVx	T2D	OVx–T2D
Histomorphometry				
Submandibular gland weight (g)	0.0413 ± 0.002	0.0523 ± 0.002^***c***^	0.0693 ± 0.002^***aaaa b***^	0.0650 ± 0.005^***aaa***^
# Acini (100 μm^2^)	13.85 ± 1.446	9.47 ± 0.619 ^***a***^	9.42 ± 0.973^***a***^	10.76 ± 0.386
Acini thickness (μm)	14.59 ± 1.186	18.17 ± 0.406 ^***c***^	22.22 ± 0.915^***aaaa b***^	20.11 ± 0.505^***aaa***^
# Acinar cells	5.30 ± 0.191	5.94 ± 0.287	6.61 ± 0.570	6.43 ± 0.275
# Striated ducts (100 μm^2^)	0.49 ± 0.042	0.54 ± 0.42	0.46 ± 0.112	0.98 ± 0.413
Striated ducts thickness (µm)	25.17 ± 1.022	22.20 ± 0.762	25.90 ± 2.782	20.41 ± 2.076
# Granular convoluted tubule	2.47 ± 0.387	2.03 ± 0.254	1.41 ± 0.160^***a***^	1.65 ± 0.204
Granular convoluted tubule thickness (µm)	22.73 ± 0.765	22.36 ± 1.279	23.64 ± 1.836	19.36 ± 1.062
Cytokines				
IL-1 (%)	1.86 ± 4.196	31.05 ± 3.774^***aaa***^	20.62 ± 4.527^***aa***^	18.83 ± 3.502^***a***^
IL-6 (%)	8.59 ± 2.542	28.34 ± 4.010^***a***^	30.18 ± 5.882^***a***^	22.33 ± 4.240
IL-10 (%)	25.00 ± 3.541	19.49 ± 4.504	9.43 ± 2.605^***a***^	8.42 ± 3.244^***a***^
IL-17 (%)	4.17 ± 0.897	23.23 ± 5.289^***a***^	11.76 ± 2.630	12.59 ± 5.044
TNF-α (%)	9.322 ± 1.534	30.94 ± 6.326^***aa***^	27.24 ± 4.083^***a***^	19.32 ± 2.185

*a* vs. control, *b* vs. OVx, *c* vs. T2D, *d* vs. OVx–T2D. *a* < 0.05, *aa* < 0.01, *aaa* < 0.005,o *aaaa* < 0.001

**Fig. 3:**
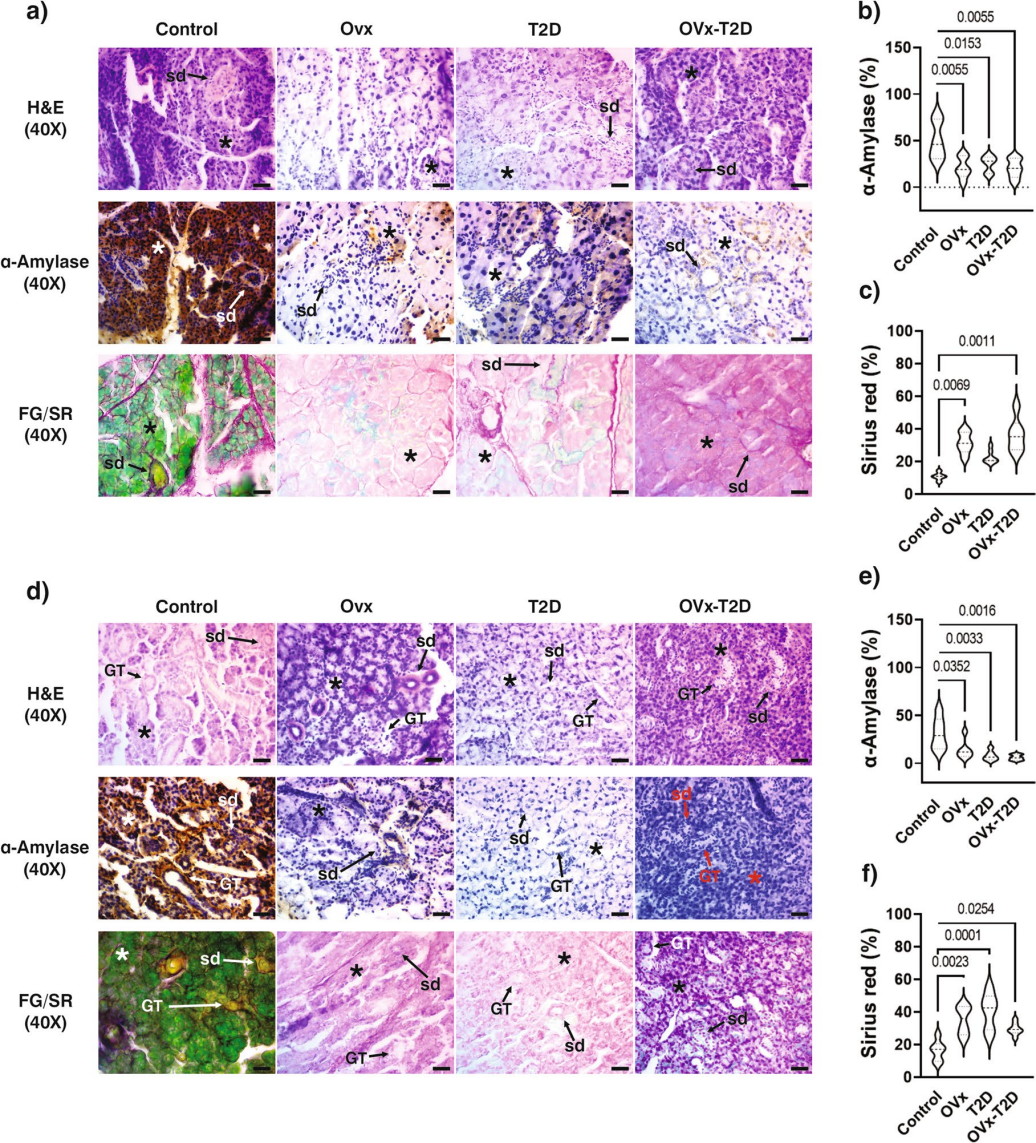
Estrogen deficiency in healthy or T2D female mice promotes the hypertrophy of acini, glandular fibrosis, and decreased expression of α-amylase in the parotid and submandibular glands. The results obtained from the four experimental groups are shown for **a**, **b**, **c** histological analysis of the parotid glands, where **a** shows representative micrographs of H and E-stained sections, of α-amylase expression obtained by IHC and fast green/Sirius red double-stained sections, **b** the percentage of α-amylase expression, and **c** the percentage of Sirius red expression. **d**–**f**) Histological analysis of submandibular glands, where **d** representative micrographs of H&E-stained sections, α-amylase expression obtained by IHC, and fast green/Sirius red double-stained sections; **e** the percentage of α-amylase expression; and **f** the percentage of Sirius red expression. The graphs present the means ± SEMs. The brackets express statistical significance, and the p value is shown. All micrographs were taken at 400 × magnification. In the micrographs, * indicates the acini, *sd* indicates the striated ducts, *GT* indicates granular convoluted tubules, and the scale bar represents 25 µm

Histomorphometric analysis of the parotid glands revealed that, compared with the control group, the OVx, T2D, and OVx–T2D groups presented a significant decrease in the number of acini (Fig. 3a and Table 1), a significant increase in the thickness of the acini (Fig. 3a and Table 1), a significant increase in the percentage of Sirius red (Fig. 3a and c), and a significant decrease in the expression of α-amylase (Fig. 3a and b). We noted no significant differences in the number and thickness of striated ducts in the four groups (Table 1). No significant differences were found between OVx, T2D, and OVx–T2D groups for any of the parameters assessed.

Histomorphometric analysis of the submandibular glands revealed that, compared with the control group, the OVx, T2D, and OVx–T2D groups presented a significant decrease in the number of acini (Fig. 3d and Table 2), a significant increase in the thickness of the acini (Fig. 3d and Table 2), a significant increase in the percentage of Sirius red (Fig. 3d and f), and a significant decrease in the expression of α-amylase (Fig. 3d and e). No significant differences among OVx, T2D, and OVx–T2D groups were found for these same parameters.

With respect to the ducts, the number and thickness of striated ducts and thickness of granular convoluted ducts did not differ remarkably among the four groups, although the number of granular convoluted ducts in the T2D group was significantly lower (Table 2).

### Estrogen deficiency favors the inflammatory microenvironment in the parotid and submandibular glands in healthy or T2D female mice

**Fig. 4:**
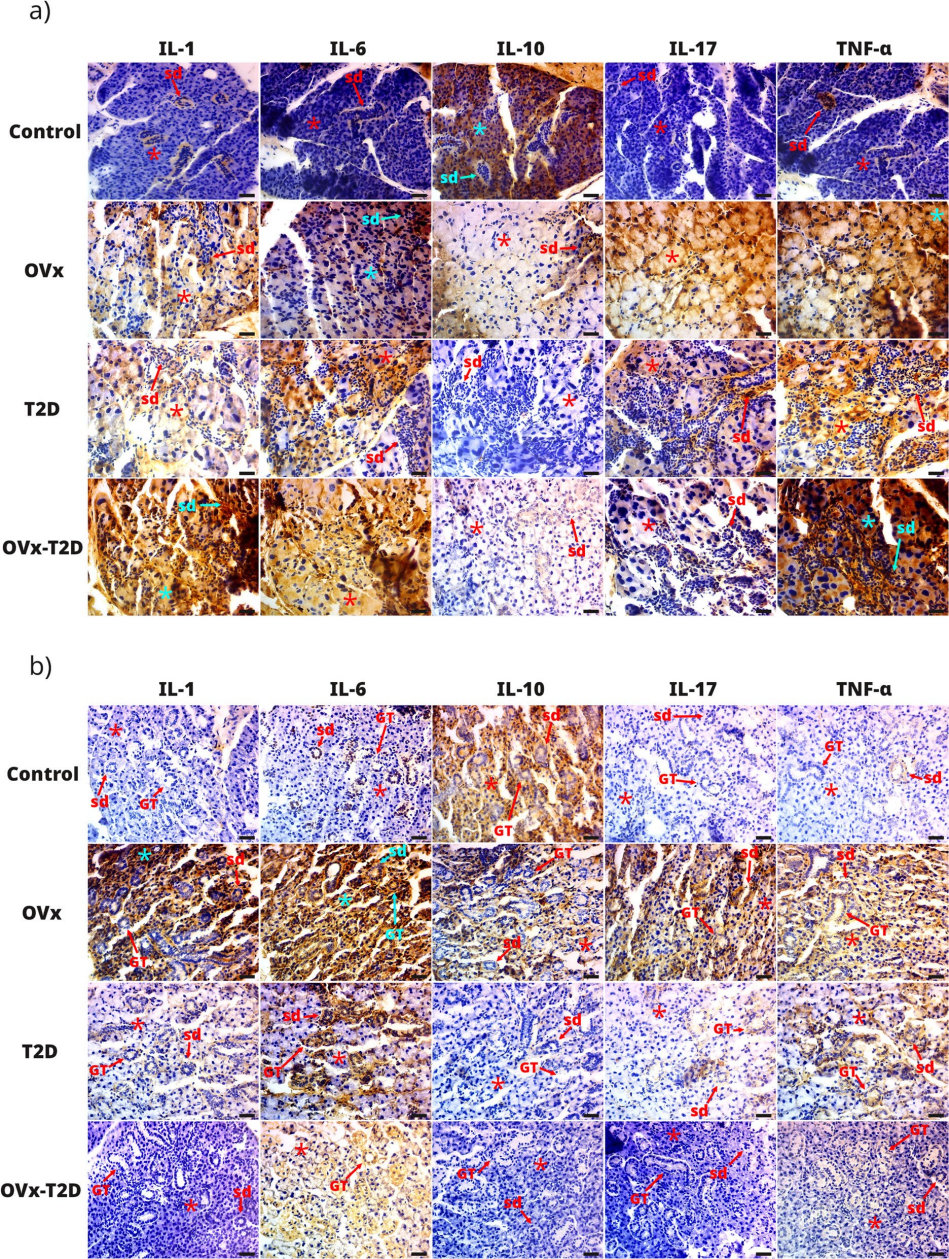
Estrogen suppression favors the inflammatory microenvironment in the parotid and submandibular glands in healthy or T2D female mice. The results of cytokine expression analysis of the parotid and submandibular salivary glands obtained by immunohistochemistry. **a** Representative micrographs from the same mouse and region per group of immunohistochemically stained histological sections of the parotid gland obtained at 400 × magnification for immunodetection of IL-1β, IL-6, IL-10, IL-17, and TNF-α. **b** Representative micrographs of histological sections of submandibular glands from the same mouse and region from each group obtained at 400 × magnification for immunodetection of IL-1β, IL-6, IL-10, IL-17, and TNF-α. * indicates the acini, *sd* indicates the striated ducts, *GT* indicates granular convoluted tubules, and the scale bar represents 25 µm

Analysis of cytokine expression in the parotid glands revealed low basal expression of IL-1, IL-6, IL-17 and TNF-α in the control group, mainly in the striated ducts, and high basal expression of IL-10, which we observed in the acinar and ductal cells and in the glandular stroma (Fig. 4a and Table 1). In comparison, the OVx, T2D and OVx–T2D groups presented significant increases in the expression of IL-1, IL-6, IL-17 and TNF-α in acinar, ductal and glandular stromal cells; furthermore, we observed a significant decrease in the expression of IL-10 in these three groups compared with that in the control group (Fig. 4a and Table 1). No differences in expression of any of the cytokines analyzed were noted, comparing the OVx, T2D and OVx–T2D groups (Table 1).

The analysis of cytokine expression in the submandibular glands revealed that the control group presented low basal expression of IL-1, IL-6, IL-17 and TNF-α, mainly in the glandular stroma, and high basal expression of IL-10 in acinar and ductal cells and in the glandular stroma (Fig. 4b and Table 2). When compared with the control group, the OVx group presented significant increases in the expression of IL-1, IL-6, IL-17 and TNF-α (Table 2) in acinar and ductal cells and in the glandular stroma (Fig. 3b); however, we did not observe changes in the expression of IL-10 (Fig. 4b and Table 2). When compared with those in the control group, the expression of IL-1, IL-6, and TNF-α in the T2D group were significantly greater (Fig. 4b and Table 2). These cytokines were expressed mainly in ductal cells and in the glandular stroma (Fig. 4b); in addition, we observed a significant decrease in the expression of IL-10 (Fig. 4b and Table 2). Compared with the control group, the OVx–T2D group presented significant increases in IL-1 and IL-6 (Table 2), where IL-1 expression was observed mainly in acinar cells and IL-6 in acinar and ductal cells and in the glandular stroma (Fig. 4b). We did not observe significant differences in the other cytokines compared with those in the control group (Table 2). Expression of each of the cytokines measured did not differ between the OVx, T2D, and OVx–T2D groups (Table 2).

## Discussion

In this work, we demonstrate that T2D causes estradiol deficiency, and that this deficiency contributes significantly to the development of hyposalivation, alterations in saliva quality, the development of histomorphometric alterations, fibrosis, decreased α-amylase and the generation of an inflammatory microenvironment in the salivary glands in T2D.

Our results demonstrated that T2D induction in female mice causes a decrease in 17β-estradiol, providing consistency with previous reports that have shown a decrease in estradiol and the development of premature postmenopause in women with T2D [3, 4, 31]. These findings are very interesting, as they demonstrate that the development of metabolic diseases can directly impact sex hormone levels, which, in turn, could accelerate and aggravate the development of T2D even in young women, which is becoming more common [32]. In fact, the loss of the protective action of estrogens in energy metabolism and their immunoregulatory role predisposes to increases the severity of metabolic disorders such as obesity and T2D [33–37], which is consistent with our results, where the OVx group had a similar increase in body weight to the T2D group despite consuming a nonhypercaloric diet, and the OVx–T2D group had higher body weight and blood glucose values than did the T2D alone group.

Although some studies have associated hyposalivation in T2D individuals with poor glycemic control [38, 39]*,* others have not reported an association with glycemic control [10]. Our results suggest that estrogen deficiency in T2D may be an important mechanism, since we observed that all three experimental groups with decreased estradiol (OVx, T2D, and OVx–T2D) had a significant decrease in stimulated salivary secretion. Some studies have reported that salivary flow rates depend on the estrogen concentration [17]. In fact, postmenopausal women have lower salivary flow rates than premenopausal women do, and hyposalivation is now considered a common symptom of postmenopause [40]; a higher prevalence of hyposalivation has been observed in diabetic women than in men [10]. Therefore, in T2D, reduced salivary flow in women may be related to estrogen deficiency.

Furthermore, we observed that all three groups with decreased estradiol had changes in saliva quality. It has been reported that T2D and decreased estrogen due to menopause can reduce salivary pH [41–43], which, at least in T2D, has been linked to increased microbial activity or a decrease in bicarbonate ions [43] and promotes the development of periodontitis, caries and other oral infections [44]. Similarly, changes in saliva density are related to the content of mucins, which help lubrication, the integrity of the oral mucosa, swallowing and phonation [45]. Regarding salivary protein content, the T2D and OVx–T2D groups presented normal total protein concentrations, whereas the OVx group presented a decrease in total protein concentration, which is consistent with previous reports that DT2 increases the salivary protein content [46, 47] and is likely due to changes in the concentrations of different secreted proteins; since it has been reported that T2D causes a decrease in amylase and mucin levels, whereas the levels of carbamoylphosphate synthetase 1, heat shock protein 60, and apoptosis-related proteins such as BAX and Caspase 3 increase [48]. It is therefore possible that estrogen deficiency due to T2D favors apoptosis in salivary acinar cells, which would explain the decrease in the number of acini that we observed.

The decrease in the amount of saliva secreted and in the quality parameters that we analyzed may be due to the histological damage evidenced by the decrease in the number of acini, acinar hypertrophy, and fibrosis and the decrease in α-amylase, which seems to be dependent on the inflammation mediated by estrogen deficiency. Estrogen deficiency in ovariectomized rats promotes the expression of caspase 3, inducing cellular apoptosis and glandular atrophy, in addition to mitochondrial defects, which are counteracted by the exogenous administration of estradiol [49]. Furthermore, fibrosis in the salivary glands is considered a predominant pathological manifestation in the pathophysiology of salivary gland diseases, and this fibrosis can induce atrophy and cell death and favor the loss of salivary gland function [50]. It has been proposed that estrogen deficiency may induce fibrosis in the salivary glands, as observed in ovariectomized mice [51].

The increase of inflammation mediators is related to the salivary gland fibrosis. The increased TNF-α has been reported to lead to increased apoptosis in rat submandibular gland acinar cells (SMG-C6) and human submandibular gland tissue samples [52]. A transgenic model in which TNF-α was overexpressed in the salivary glands of mice, induced an increase in fibrosis, lymphoid infiltration, apoptosis and the production of other inflammatory cytokines such as IL-1 and IL-6 in the submandibular glands. Moreover, in women with hyposalivation, an increase in the expression of TNF-α was observed in biopsies of the parotid gland [53], This coincides with our results in where we observed an increase of IL-1, IL-6, IL-17 and TNF-α and the development of fibrosis, the decrease in acini and the glandular hypofunction.

The inflammation in the salivary glands that we observed seems to be due to the decrease in estrogens, since the three groups that had a decrease in estradiol showed an increase in inflammatory cytokines, with a decrease in IL-10. This is plausible given that estrogens are recognized as important immunomodulatory hormones that suppress the production of IL-1, IL-6, and TNF-α by different immune cell populations and the induction of IL-10 and T regulatory cells maintenance [54], which coincides with the increase in inflammatory cytokines and the decrease in IL-10 in both salivary glands.

Therefore, our results indicate that decreased estrogen in female mice with T2D or OVx contributes significantly to the development of hyposalivation, histomorphometric alterations and fibrosis of the parotid and submandibular glands by promoting an inflammatory microenvironment in the parotid and submandibular glands of female mice.

## Supplementary Information

Below is the link to the electronic supplementary material.

Supplementary file1 (DOCX 596 KB)

## Data Availability

The datasets used and/or analyzed during the current study are available from the corresponding author on reasonable request.
