# Supplementary material for: Estrogen deficiency due to type 2 diabetes induced hyposalivation in female mice by promoting inflammation in the salivary glands
Source: Odontology. 2025 Jul 29;114(2):788–99. doi: 10.1007/s10266-025-01158-6 (PMC13053413; doi:10.1007/s10266-025-01158-6)
Supplement: Supplementary file 1 — Supplementary file1 (DOCX 596 KB) [file 10266_2025_1158_MOESM1_ESM.docx]

**SUPPLEMENTARY MATERIAL**

**Title:** Estrogen deficiency due to type 2 diabetes induces hyposalivation in female mice by inducing inflammation in the salivary glands.

**Authors:** Cifuentes-Mendiola S.E.**^1^***; García-Hernández A.L.**^1^**, Cruz-Mendoza N.**^1^**, Cruz-García I.X.**^1^**


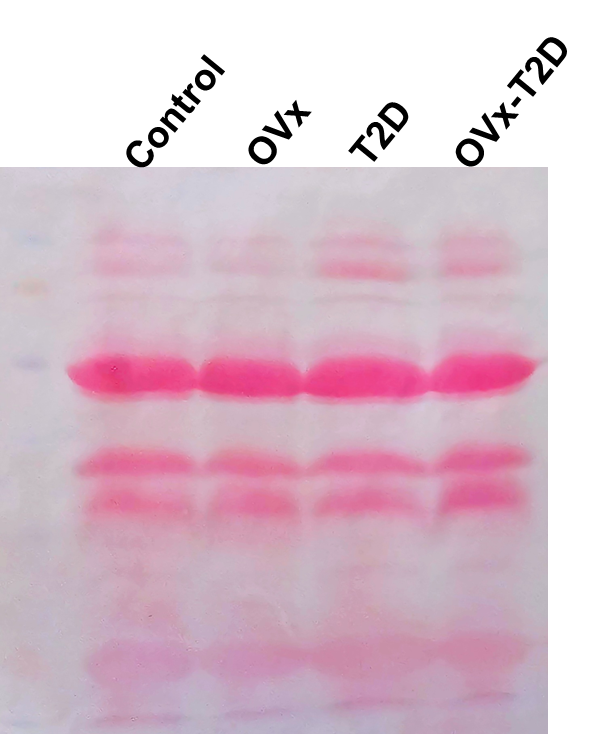


**Supplementary Figure 1** Representative photograph of the western blot of saliva proteins from the different experimental groups stained with Ponceau red.


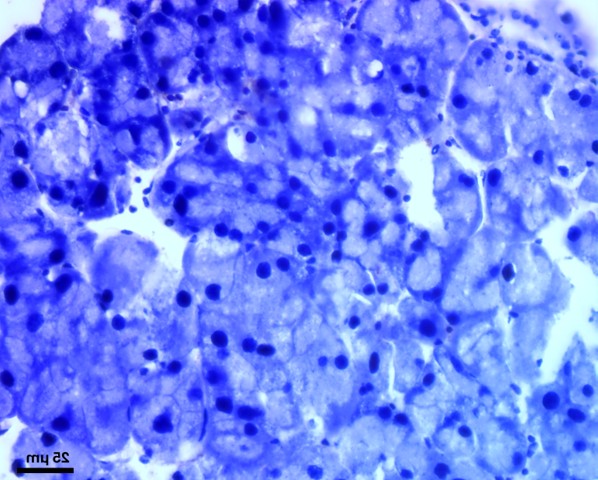


**Supplementary Figure 2** Representative photograph of the negative control for IHC obtained with the SS Polymer-HRP/DAB Detection Kit, according to the manufacturer's specifications.
